# Conductive Fe_3_O_4_ Nanoparticles Accelerate Syntrophic Methane Production from Butyrate Oxidation in Two Different Lake Sediments

**DOI:** 10.3389/fmicb.2016.01316

**Published:** 2016-08-22

**Authors:** Jianchao Zhang, Yahai Lu

**Affiliations:** College of Urban and Environmental Sciences, Peking UniversityBeijing, China

**Keywords:** syntrophy, methane, methanogenesis, direct interspecies electron transfer, lake sediments, Fe_3_O_4_, butyrate

## Abstract

Syntrophic methanogenesis is an essential link in the global carbon cycle and a key bioprocess for the disposal of organic waste and production of biogas. Recent studies suggest direct interspecies electron transfer (DIET) is involved in electron exchange in methanogenesis occurring in paddy soils, anaerobic digesters, and specific co-cultures with *Geobacter*. In this study, we evaluate the possible involvement of DIET in the syntrophic oxidation of butyrate in the enrichments from two lake sediments (an urban lake and a natural lake). The results showed that the production of CH_4_ was significantly accelerated in the presence of conductive nanoscale Fe_3_O_4_ or carbon nanotubes in the sediment enrichments. Observations made with fluorescence *in situ* hybridization and scanning electron microscope indicated that microbial aggregates were formed in the enrichments. It appeared that the average cell-to-cell distance in aggregates in nanomaterial-amended enrichments was larger than that in aggregates in the non-amended control. These results suggested that DIET-mediated syntrophic methanogenesis could occur in the lake sediments in the presence of conductive materials. Microbial community analysis of the enrichments revealed that the genera of *Syntrophomonas*, *Sulfurospirillum*, *Methanosarcina*, and *Methanoregula* were responsible for syntrophic oxidation of butyrate in lake sediment samples. The mechanism for the conductive-material-facilitated DIET in butyrate syntrophy deserves further investigation.

## Introduction

Methane (CH_4_) is not only an important greenhouse gas, but also a well-known component of biogas, which is used as fuel. Methanogens, which obtain energy for growth by utilizing a few simple substrates (such as H_2_, formate, and acetate), are widespread in anoxic environments (Thauer et al., 2008). Because methanogens are able to use only a limited number of substrates, they depend on syntrophic cooperation for decomposing complex organic compounds in anoxic environments such as rice paddies, wetlands, fresh water sediments, anaerobic bioreactors, and the intestinal tracts of animals and insects (Stams and Plugge, 2009; Sieber et al., 2012).

It is well recognized that interspecies electron transfer (IET) via H_2_ or formate diffusion occurs between proton-reducing acetogens and methanogens. IET has been considered a bottleneck step in the methanogenic decomposition of complex organic substances. Recently it was proposed that direct interspecies electron transfer (DIET) is an alternative to the interspecies H_2_/formate transfer in syntrophic methanogenesis (Morita et al., 2011; Rotaru et al., 2014b). The DIET-mediated syntrophic methanogenesis has been demonstrated in co-cultures of *Geobacter metallireducens* and *Methanosaeta* (Rotaru et al., 2014b) or *Methanosarcina* species (Rotaru et al., 2014a). The outer surface c-type cytochromes and electrically conductive pili are considered to play a role in mediating DIET when *Geobacter* species are involved (Lovley, 2012; Shrestha and Rotaru, 2014).

In addition to biogenic mediators, naturally occurring conductive or semiconductive materials can also facilitate DIET in syntrophic methanogenesis. Magnetite (Fe_3_O_4_), a mineral containing Fe(II) and Fe(III) in a ratio of 1:2 which originates either biologically (microbially) or geologically is ubiquitous in environments like weathered soils and freshwater sediments (Skomurski et al., 2010; Liu et al., 2015). This mineral is electrically conductive. Kato et al. (2012) showed that the supplementation of Fe_3_O_4_ in rice paddy soil significantly accelerated CH_4_ production from ethanol and acetate, and that *Geobacter* and *Methanosarcina* species were significantly enriched (Kato et al., 2012). Several other studies suggested that the supplementation with artificial electrically conductive materials (e.g., granular activated carbon, biochar) could also promote DIET-mediated syntrophic methanogenesis in defined co-cultures of *Geobacter* with *Methanosarcina* species (Liu et al., 2012; Chen et al., 2014; Rotaru et al., 2014a). A recent study suggested that magnetite could substitute for the pilus-associated cytochrome OmcS of *G. sulfurreducens* to mediate cell-to-cell electron transfer (Liu et al., 2015). Using of the natural or artificial nanomaterials as electron conductors to accelerate syntrophic CH_4_ production may help microbes to save energy for the biosynthesis of the extracellular, biological and electrical connections.

Butyrate is one of the most important intermediates in the transformation of complex organic matter to CH_4_. DIET was not expected to occur in the syntrophic oxidation of butyrate because recognized butyrate syntrophs were not known to possess genetically determined extracellular electron transfer components (Sieber et al., 2012). But a recent study showed that the syntrophic production of CH_4_ from butyrate oxidation in paddy soil enrichment was significantly accelerated in the presence of nanoFe_3_O_4_, which was thought to be due to DIET activity (Li et al., 2015). Anaerobic freshwater lake sediments represent important methanogenic environments (Bastviken et al., 2004). The syntrophic enrichments were prepared from two lake sediments (an urban and a natural lake) with butyrate as the sole substrate in the presence of nanoFe_3_O_4_. We determined the effect of nanoFe_3_O_4_ on syntrophic methanogenesis and the corresponding community structure in lake sediment samples and attempted the identification of the organisms responsible for DIET. Furthermore, carbon nanotubes (CNTs) were used to determine if artificial electrically conductive nanomaterials could be substituted for nanoFe_3_O_4_.

## Materials and Methods

### Collection of Lake Sediments

Two lake sediment samples were collected at the depth of 0–15 cm from the sediment surface: one from Weiming Lake (WML), a pond on the campus of Peking University (39°59′36″N 116°18′12″E) and the other from Erhai Lake (EHL), which is located on the Yungui plateau within Yunnan Province in the southwestern China (26°01′N 100°03′E). WML has an area of about 3 ha and an average water depth of 1.5 m. The lake freezes temporally in winter. The pond is eutrophic. A sediment sample from it was analyzed. It contained 6.25% total organic carbon, 0.38% total nitrogen, and its pH was 7.78. The EHL is the second largest freshwater lake (256.5 km^2^) on Yungui plateau. It is situated at an altitude of 1934 m and has average water depth of 10.5 m. The water source of EHL is rainfall and the melt water from ice and snow. The sediment sample from EHL contained the 2.40% total organic carbon and 0.27% total nitrogen. Its pH was 7.20 (Wu et al., 2015). Sediment samples were obtained by scraping the sediment surface (0–15 cm) with a 3-liter sampler.

### Preparation of Magnetite Nanoparticles and Multi-Walled CNTs

The nanoFe_3_O_4_ was synthesized via a conventional aqueous co-precipitation method (Kang et al., 1996). Briefly, 2 g FeCl_2_ and 5.2 g FeCl_3_ were dissolved in 0.4 N HCl solution and 1.5 N NaOH solution was added drop-wisely under vigorous mechanical agitation. Upon the addition of NaOH, precipitation of a black precipitate of magnetite was observed. The precipitate was separated with an external magnet, and then washed repeatedly with deionized water until the supernatant wash water was neutral (pH 7). Commercially available multi-walled CNTs (MWCNTs; 10–20 nm in diameter and 10–30 μm in length) were purchased from DK Nano Technology Co. (Beijing, China). MWCNTs were carboxylic acid functionalized (2% -COOH by weight) and were >98% pure. A stock suspension of 10% (w/v) MWCNTs in the water was used for the experiment.

### Enrichment Cultivation

Approximately 0.5 g (WML) or 5 g (EHL) fresh sediment (microbial inocula for first enrichments) were put into sterile 120 serum bottles filled with 50 ml of HEPES-buffered (30 mM, pH 7) medium supplemented with sodium butyrate to a final concentration of 10 mM. The basal medium consisted of 0.4 g MgCl_2_, 0.1 g CaCl_2_, 0.1 g NH_4_Cl, 0.2 g KH_2_PO_4_, 0.5 g KCl, and 0.0005 g resazurin per liter of distilled water (Lü and Lu, 2012). Supplements of NaHCO_3_, NaS, vitamin, trace element solutions, and Se/W solution followed the protocol described previously (Lü and Lu, 2012). The cysteine was excluded in the medium to avoid the possible effect of electron shuttle molecules (Kaden et al., 2002). All the bottles were sealed with butyl stoppers and aluminum crimp caps. The enrichments were incubated statically in the dark at 30°C under the atmosphere of N_2_/CO_2_ [80:20 (V/V)] in the headspace of each bottle.

The first and second enrichments consisted of two treatments: (1) nanoFe_3_O_4_ were supplemented from the stock solution to give the final concentration of 10 mM as Fe atom; and (2) CK (no-amendment control). When the CH_4_ production from the Fe_3_O_4_ treatment approached a plateau, inculum from nanoFe_3_O_4_ treatment (4% inoculum) were transferred to medium as described above. In the third and fourth enrichment transfers, three treatments were prepared: control without supplements (CK), Fe_3_O_4_ addition at a final concentration of 10 mM as Fe atom and MWCNTs addition at a final concentration of 0.5% (w/v). After the third transfer, each culture was examined microscopically and subjected to molecular phylogenetic analysis. The experiment was carried out in triplicate. The design of experiment is shown in the **Supplementary Figure 1**.

**FIGURE 1:**
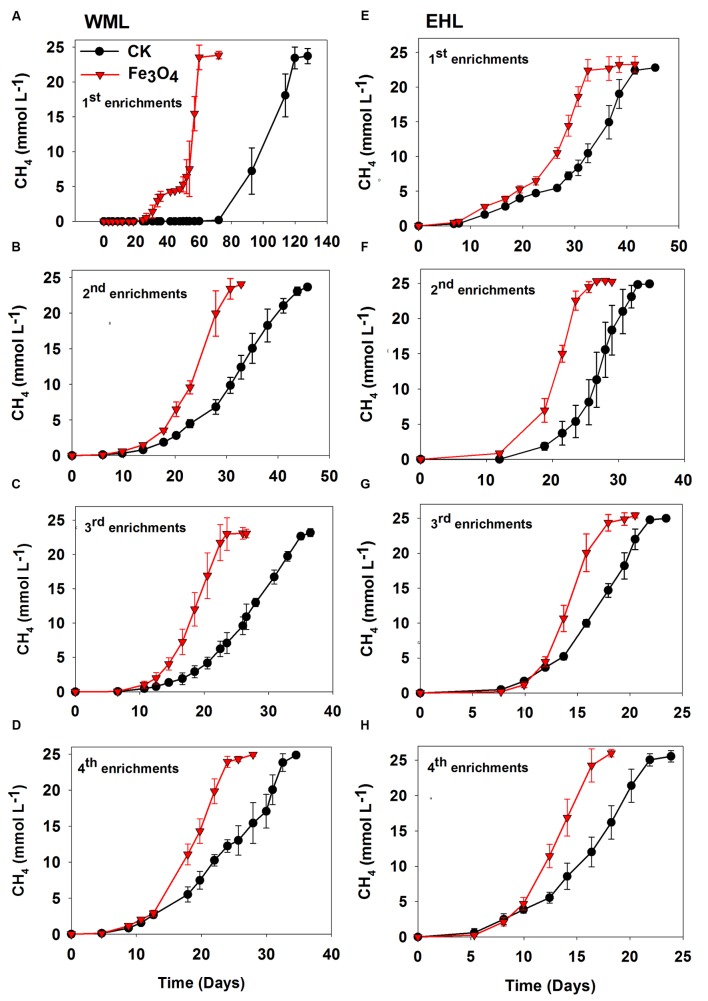
**Effects of conductive Fe_3_O_4_ nanoparticles on the production of CH_4_ in the enrichments from Weiming Lake sediment, WML **(A–D)**, and Erhai Lake sediment, EHL **(E–H)**.** Error bars represent the standard deviation of replicate experiments.

### Chemical Analyses

Gas samples (100 μl) were regularly taken from the headspace using a Pressure-Lok precision analytical syringe (Bation Rouge, LA, USA). The CH_4_ concentration was analyzed using GC-7890 gas chromatograph (Agilent Technologies, USA) equipped with flame ionization detector (FID). The detection limit was 50 ppmv. For the analysis of organic acids including butyrate and acetate, 0.2 ml of culture medium was sampled with sterile syringes. Butyrate and acetate were analyzed by injecting filtered (0.22 μm porosity) liquid sample into a gas chromatograph equipped with FID detector (injection, detection, and column temperatures were 200, 250, and 120°C, respectively). We used a 30 m capillary column (DB-Waxetr, 0.53 mm i.d., 1 μm film thickness) to separate organic acids with a split ratio of 10:1. The HCl-extractable Fe(III) and Fe(II) were determined by the ferrozine technique as described elsewhere (Lovley and Phillips, 1986).

### Microscopy

The cells in the mid-log phase were collected by a syringe fixed with 2.5% (wt/vol) glutaraldehyde in phosphate-buffered saline and sequentially dehydrated with ethanol (20, 40, 60, 80, 95, and 100% (v/v) ethanol, 10 min for each treatment). The dried samples were coated with platinum and imaged using scanning electron microscope (FEI NanoSEM 430).

Fluorescence *in situ* hybridization (FISH) analysis was performed on 4% paraformaldehyde-fixed samples according to a procedure described elsewhere (Moter and Göbel, 2000). Oligonucleotide probes specific for bacteria (Cy3-labeled EUB338mix probes) and archaea (FITC-labeled ARC915 probe) were used in this study. The details of the probes used are available in the probebase (Loy et al., 2003). The labeled samples were visualized using epifluorescence microscopy (Axio imager D2, ZEISS).

### Analysis of Microbial Community Composition

The triplicate cultures growing in the presence of nanoFe_3_O_4_ in the fourth transfer were used to extract microbial DNA. Prior to extraction, sonication treatment was conducted to separate the microbial cells from Fe_3_O_4_ nanoparticles. Total DNA was extracted using the FastDNA SPIN Kit (MP Biomedicals, USA) according to the manufacturer’s protocol. Prior to PCR amplification, the DNA extracts from each replicate were mixed in equal amount. DNA extracts were stored at -20°C.

Bacterial and archaeal clone libraries were constructed from the WML enrichment. PCR amplification was performed using the primer pairs of Ba27f/907r for bacteria and Ar109f/Ar915r for archaea. PCR products were purified and clone library analyses were conducted as described previously (Peng et al., 2008). One hundred bacterial clones and one hundred archaeal clones were retrieved from the WML enrichment. Sequences of the clone libraries were analyzed by defining operational taxonomic units (OTU), in which representative sequences from each OTU was defined by 97% sequence identity. The closest matching sequences were identified by searching with the BLAST program in the NCBI database. Phylogenetic trees were constructed using MEGA 6.0 with the neighbor-joining method. The 16S rRNA gene sequences obtained in this study were deposited in the GenBank databases under accession numbers from KU577285 to KU577288.

Because relatively diverse cell morphologies were observed in EHL enrichment, the high-throughput sequencing was used for the microbial community analysis. After DNA extraction, the qualified DNA was sent to Sangon Biotech Company (Shanghai, China) for amplicon sequencing using the Illumina Miseq 2 × 300 bp platform (San Diego, CA, USA). The V3–V4 universal primers 314F (CCTACGGGNGGCWGCAG) and 805R (GACTACHVGGGTATCTAATCC) with sample-specific barcodes were used for interrogating bacterial communities. A nested PCR amplification, with the primer sets of 340F (CCCTAYGGGGYGCASCAG) and 1000R (GGCCATGCACYWCYTCTC) for the first round (25 cycles), and 349F (GYGCASCAGKCGMGAAW) and 806R (GGACTACVSGGGTATCTAAT) for the second round (25 cycles) was conducted to amplify the archaeal 16S rRNA genes. More than 30,000 sequences were obtained from each sample. Sequences were clustered into OTUs using UCLUST software^1^ with a similarity threshold of 97%. The Ribosomal Database Project (RDP) classifier was used to assign the taxonomic data to each representative sequence. Raw sequencing reads obtained in this study have been deposited in the NCBI SRA with the accession number SRP068809 and SRP068811.

## Results

### CH_4_ Production

Four enrichment transfers were performed for each sediment sample. In all transfers, we observed consistently increased rates of CH_4_ production in the presence of nanoFe_3_O_4_ (**Figure 1**). In all incubations, about 25 micromoles of CH_4_ per liter were produced from 10 micromoles of butyrate added. Acetate showed only intermediate accumulation (**Supplementary Figure 2**). This result indicated that methanogenesis in the enrichments followed the stoichiometry of complete conversion of butyrate to CH_4_ and CO_2_. For the WML sediment, the first enrichment was inoculated with 0.5 g of fresh sample into 50 ml medium. CH_4_ production displayed a substantial lag (**Figure 1A**), but this lag was markedly shortened in the presence of nanoFe_3_O_4_ (27 days versus 72 days in the control). The conversion of butyrate to CH_4_ was completed at 61 days in nanoFe_3_O_4_ compared with 119 days in the control. The lag phase was reduced significantly in the second and later transfers (**Figures 1B–D**). For the oligotrophic EHL, the first enrichment was inoculated with 5 g of fresh sediment. The initiation of CH_4_ production was faster than with the WML sediment. The stoichiometric conversion of butyrate in the first transfer of EHL enrichment was obtained at 32 days in the presence of nanoFe_3_O_4_ compared with 42 days in the control (**Figure 1E**). For both sediments, CH_4_ production was faster in the third and fourth enrichments compared with the first and second enrichments. The calculated maximum rate of CH_4_ production during the exponential growth phase was about 60–90% (WML) and 34–56% (EHL) greater in the presence of nanoFe_3_O_4_ than in the control (**Supplementary Figure 3**).

**FIGURE 2:**
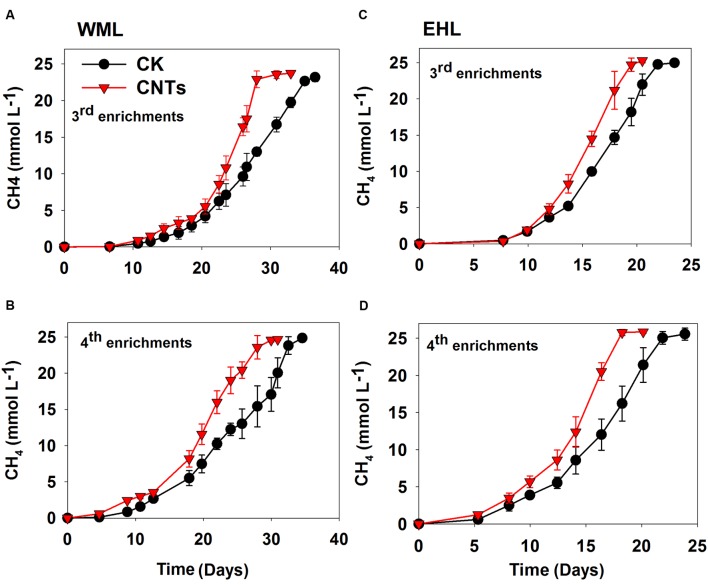
**Effects of conductive Multi-walled CNTs on the production of CH_4_ in the third and fourth enrichments from WML **(A,B)** and EHL **(C,D)****.

In the third and fourth transfers, MWCNTs were applied in parallel. The transfers involved inocula from previously nanoFe_3_O_4_-amended enrichments. The addition of MWCNTs accelerated CH_4_ production in all of the enrichments (**Figure 2**). The time needed for the complete conversion of butyrate to CH_4_ and CO_2_ was significantly shortened in the presence of MWCNTs compared with the control. The maximum rates of CH_4_ production in the presence of MWCNTs were approximately 50% greater relative to the control (**Supplementary Figure 4**).

### Microscopic Observation

**FIGURE 3:**
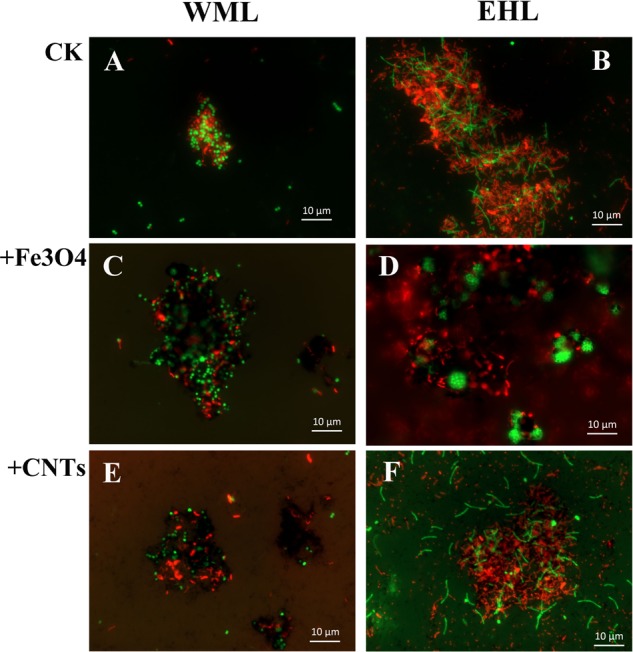
**Spatial distribution of archaeal (Arc915-FITC, green) and bacterial (EUB338mix-Cy3, red) cells identified by FISH in WML and EHL enrichments with CK, Fe_3_O_4_ and MWCNTs treatments.** (CK treatment: **A,B**; Fe_3_O_4_ treatment: **C,D**; WMCNTs treatment: **E,F**). The Fe_3_O_4_ nanoparticles and the MWCNTs appear black in the FISH images from the corresponding treatments.

The oligonucleotide probes used for FISH observations targeted bacteria and archaea. The fluorescence images revealed that numerous aggregates were formed in both WML and EHL enrichments (**Figure 3**). In many cases, it appeared that the bacterial cells formed dense cores with archaeal cells residing peripherally (**Figures 3A,B**). Most of bacterial cells were curved and rod-shapes and were morphologically similar in the WML and EHL enrichments. Archaeal cells were mostly coccoid in the WML enrichment (**Figures 3A,C,E**), whereas more of archaeal cells in the EHL enrichment were slender rods (**Figures 3B,F**). The microbial aggregates displayed distinct architectures between the control and those in nanoFe_3_O_4_ and MWCNTs treatments. In the control, the bacterial and archaeal cells in aggregates were in close physical proximity (**Figures 3A,B**). But in the nanoFe_3_O_4_ and MWCNTs treatments, there existed dark areas within aggregates (**Figures 3C–F**). These dark areas were filled with nanoFe_3_O_4_ particles and MWCNTs. The aggregates formed in the presence of nanoFe_3_O_4_ or MWCNTs seemed to exhibit greater intercellular distances on average than aggregates in the control. However, most of the cells in the presence of either nanoFe_3_O_4_ or MWCNTs were interconnected by the respective particles.

**FIGURE 4:**
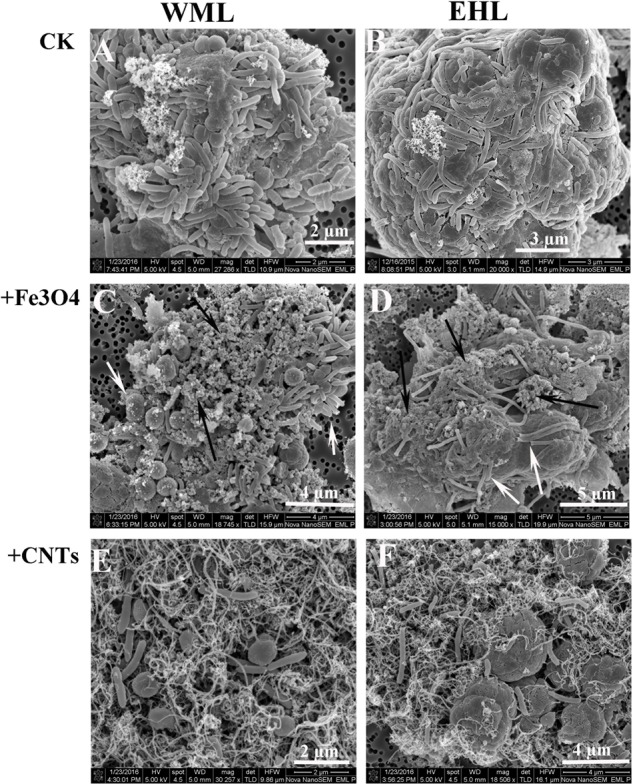
**Scanning electron micrographs (SEM) of cell aggregates or cell/material mixtures in the enrichment cultures from WML and EHL (WML: A,C,E; EHL: B,D,F).** White arrows indicate cells and the black arrows indicate Fe_3_O_4_ nanoparticles.

The scanning electron microscope (SEM) corroborated the FISH observations (**Figure 4**). **Figures 4A,B** showed that the cells were in close contact forming dense microbial aggregates in the control. The cells in the WML enrichment were coccus-shaped, rod-shaped and curved (**Figure 4A**). In the EHL enrichment, *Methanosarcina*-like cells and long slender rods were observed additionally (**Figure 4B**). The extracellular polymer-like substances were also detected in the aggregates. The cells from the nanoFe_3_O_4_ and MWCNTs treatments (**Figures 4C–F**) were less densely organized and showed larger intercellular distance on average compared with the control. However, most of these cells were closely associated with nanomaterials and formed cell/nanomaterial mixtures (**Figures 4C–F**).

### Microbial Communities

**FIGURE 5:**
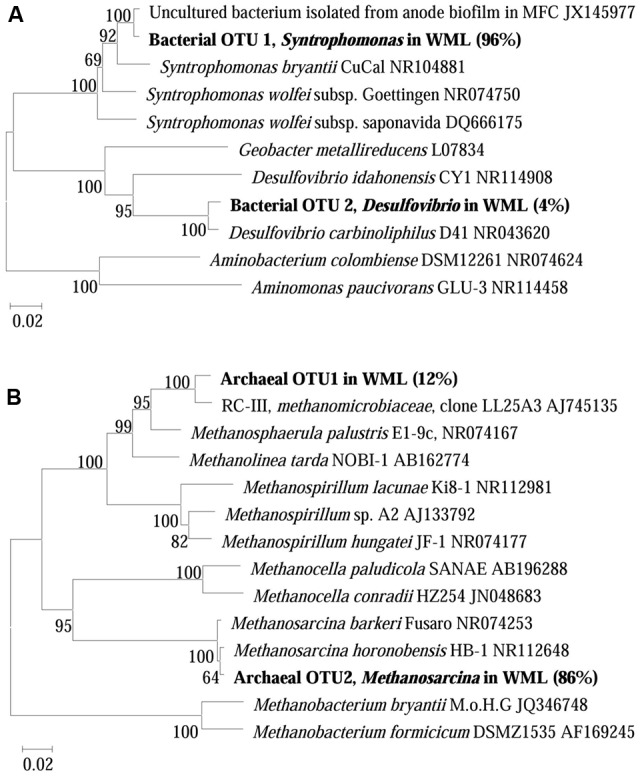
**Neighbor-joining phylogenetic tree of representative bacterial **(A)** and archaeal **(B)** 16S rRNA gene clones obtained from the fourth enrichments of WML with the addition of Fe_3_O_4_ nanoparticles.** Clones obtained in this study are indicated in boldface and their relative abundances are given in parentheses. GenBank accession numbers of reference sequences are indicated.

The composition of microbial communities in the enrichments was determined by 16S rRNA gene cloning and sequencing. We conducted the conventional cloning and sequencing with the WML enrichment and Miseq sequencing with the EHL enrichment because of the relatively more complex community in the latter. One hundred bacterial clones retrieved from the WML enrichment could be classified into two OTUs (**Figure 5A**). OTU 1 belonged to *Syntrophomonas*, accounting for 96% of the total sequences. It was closely related (99% identity) to an uncultured bacterial clone detected in an anode biofilm of a microbial fuel cell (clone BP, JX145977; Ishii et al., 2014). The closest pure culture relative was *S. bryantii* CuCal (95% identity in 16S rRNA). The remaining four sequences (OTU 2) were related to a known bacterial strain, *Desulfovibrio carbinoliphilus* D41 (98% identity in 16S rRNA; Allen et al., 2008). **Figure 5B** shows that the archaeal clone sequences (100 clones) were comprised 84% of the *Methanosarcina*-like sequences (OTU 2), with the closest pure culture relatives being *M. horonobensis* HB-1 (99.2% identity), and 16% of *Methanomicrobia*-like sequences (OTU 1) that were related to *Methanosphaerula palustris* E1-9c (94% identity).

**FIGURE 6:**
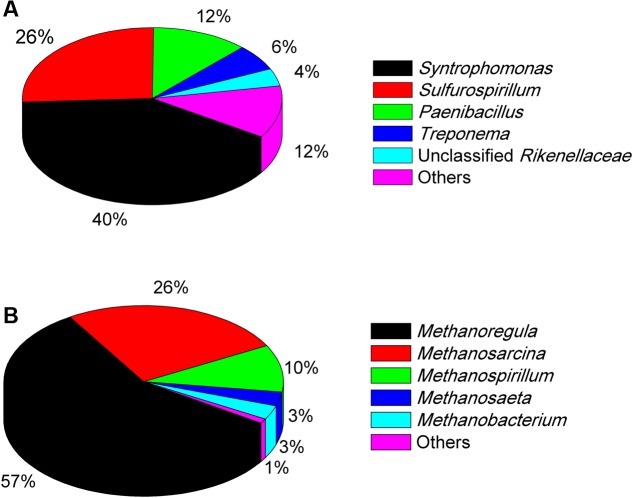
**The community composition and relative abundance of bacteria **(A)** and archaea **(B)** at genus level in the fourth enrichments of EHL as determined by Illumina Miseq sequencing.** The genus whose relative abundance was less than 3% was included in the group “Other.” More than 30,000 sequences were obtained for the bacteria and archaea phylogenetic classifications at genus level separately.

The Miseq sequencing revealed that the bacterial populations in the EHL enrichment (**Figure 6A**) were dominated by *Syntrophomonas* (40%), followed by *Sulfurospirillum* (26%) and a few other bacterial lineages *Paenibacillus*, *Treponema*, and unclassified *Rikenellaceae*. The archaeal community (**Figure 6B**) consisted mainly of *Methanoregula* (57%), *Methanosarcina* (26%) and *Methanospirillum* (10%), with a minor proportion of *Methanosaeta* and *Methanobacterium*.

## Discussion

In all of the enrichments, we found that the addition of nanoFe_3_O_4_ substantially facilitated the syntrophic production of CH_4_ from butyrate oxidation. Furthermore, the addition of MWCNTs, an artificial chemically stable nanomaterial with high electric conductivity (Baughman et al., 2002), displayed a similar stimulatory effect. These results suggest that the electric conductivity of the added nanoparticles played a key role in facilitating the syntrophic oxidation of butyrate.

Microbial aggregates were extensively formed in the sediment enrichments (**Figure 3**). It has been suggested that the formation of microbial aggregates can enhance the efficiency of interspecies H_2_ transfer due to the reduction of cell-to-cell distance for the electron-carrier diffusion (Kouzuma et al., 2015). Even though all enrichments showed microbial aggregates formation, the structure of the aggregates formed in the control and in the presence of nanoFe_3_O_4_ or MWCNTs differed from each other. The consensus of visual image estimates suggests that the presence of nanoparticles within the aggregates increased rather than shortened the intercellular distance compared with the control. If the intercellular distance were a key factor in the syntrophic interaction based on interspecies H_2_/formate diffusion, a lower rate of CH_4_ production would be expected in the presence of nanoparticles. Therefore, our results suggest that the conductive nanoparticles likely facilitated DIET in the butyrate syntrophy by forming the cell-nanomaterial-cell networks. The discrepancy between cell-to-cell distance and the syntrophic activity has also been considered to indicate the involvement of DIET in the anaerobic oxidation of CH_4_ in ocean sediments (McGlynn et al., 2015). Theoretical calculation suggested that the electron transfer rate among the syntrophic partners via direct electric conduction was substantially higher (10^6^ times) than via interspecies H_2_ diffusion (Viggi et al., 2014).

The dominant bacterial clones in both WML enrichment (96%) and EHL enrichment (40%) were affiliated with *Syntrophomonas*. The clone sequence from the WML enrichment was closely (99% identity) related to an uncultured bacterial clone retrieved from an anode biofilm of a microbial fuel cell fed with butyrate and propionate (Ishii et al., 2014). It has to be noted that the clone analysis and phylogenetic information reflected only the bacterial composition in the enrichment rather than the community in the original sediment sample. *Syntrophomonas* have been well recognized as an obligate syntroph in anaerobic oxidation of butyrate. Genomic analyses have so far not been able to identify the mechanism of extracellular electron transfer in *Syntrophomonas* (Sieber et al., 2010, 2012). In other studies, involving anaerobic membrane bioreactor (Smith et al., 2015), microbial fuel cells (Ishii et al., 2014) and microbial electrolysis cells (Zhao et al., 2016), *Syntrophomonas* were detected in the biofilms or anodic biofilms, and indicated that they was able to contribute to butyrate degradation and electricity generation.

In the EHL enrichment, we also found that *Sulfurospirillum* was enriched (26%). *Sulfurospirillum* is a sulfur-reducing bacterium able to use Fe(III), arsenate, elemental sulfur, thiosulfate, and nitrate as electron acceptors (Kodama et al., 2007), and acetate, lactate, and butyrate as electron donors (Héry et al., 2008). The present experiment was performed under methanogenic conditions. Therefore, *Sulfurospirillum* probably also directly participated in the syntrophic oxidation of butyrate in the EHL enrichment. The mechanisms deserve further investigations.

*Methanosarcina* were specifically enriched (86%) in the WML enrichment after continuous transfers in the presence of nanoFe_3_O_4_. In the EHL enrichment the hydrogenotrophic *Methanoregula* dominated (accounting for 57%) with *Methanosarcina* being the second major methanogens (26%). Methanogens serve as the syntrophic partner utilizing the electrons released from syntrophic bacteria. The molecular mechanisms of the involvement of methanogens in DIET remain unknown (Kouzuma et al., 2015). However, pure cultures of *Methanosaeta harundinacea* and *Methanosarcina barkeri* have been used in the construction of syntrophic co-cultures with *Geobacter*, which performed DIET in CH_4_ production from ethanol (Liu et al., 2012; Rotaru et al., 2014a,b). We showed earlier that *Methanosarcinaceae*, *Methanocellales*, and *Methanobacteriales* were possibly involved in DIET in paddy soil (Li et al., 2015). Other studies showed that *Methanococcus maropaludis*, *Methanobacterium palustre*, and *Methanothermobacter* spp. were capable of accepting electrons in a more direct way from cathode electrodes than from H_2_ in the electrochemical systems (Cheng et al., 2009; Lohner et al., 2014; Fu et al., 2015). In an electrochemical study, *Methanoregula* were found to be specifically enriched when the applied voltage was set lower than the threshold for H_2_ production, indicating that *Methanoregula* produced CH_4_ via cathode reaction: CO_2_ + 8H^+^ + 8e^-^ → CH_4_ + 2H_2_O (Chen et al., 2016). Thus, the extracellular electron uptake may occur with various methanogens. More studies are needed to elucidate the mechanisms of direct extracellular electron uptake by methanogens.

Based on the origin of mediators, DIET can be categorized into two forms, DIET of biological origin, which employs outer-membrane c-type cytochromes and pili or exudation of molecular redox shuttles, and DIET of environmental origin, which utilizes electron-conductive material of natural origin and/or the artificial supplements (Shrestha and Rotaru, 2014). The DIET of biological origin has been intensively studied using the defined co-cultures with *Geobacter* spp. (Lovley, 2012; Kouzuma et al., 2015). However, DIET of environment origin has been less understood. Utilization of DIET of environment origin shall confer an ecological advantage to microbes using DIET because biosynthesis of molecular conduits is not needed (Kato et al., 2012; Li et al., 2015). Furthermore, biological mediators are probably not ubiquitous among microorganisms, while the conductive-semiconductive minerals have widespread occurrence in nature. Additional studies shall clarify the significance of DIET of environment origin in methanogenesis and carbon cycling in various environments.

## Author Contributions

JZ and YL devised the study, JZ conducted the experiments and analyses, all the authors contributed to data interpretation and writing of the manuscript.

## Conflict of Interest Statement

The authors declare that the research was conducted in the absence of any commercial or financial relationships that could be construed as a potential conflict of interest.

## Footnotes

1 http://www.drive5.com/uclust
