# Supplementary material for: Conductive Fe3O4 Nanoparticles Accelerate Syntrophic Methane Production from Butyrate Oxidation in Two Different Lake Sediments
Source: Front Microbiol. 2016 Aug 22;7:1316. doi: 10.3389/fmicb.2016.01316 (PMC4992681; doi:10.3389/fmicb.2016.01316)
Supplement: Supplementary file 1 [file Data_Sheet_1.PDF]

Supplementary Material

Conductive Fe_3O_4 nanoparticles accelerate syntrophic methane production from butyrate oxidation in two different lake sediments

Jianchao Zhang, Yahai Lu*

College of Urban and Environmental Sciences, Peking University, Beijing 100871, China

*** Correspondence:** Yahai Lu: luyh@pku.edu.cn

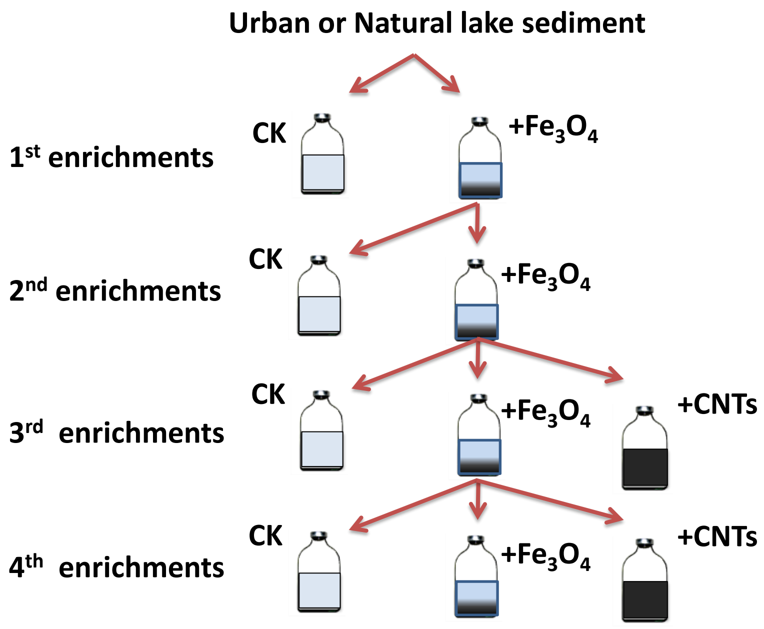

Supplementary figure 1. Schematic diagram of the experiment design with different treatments and continuous transfers

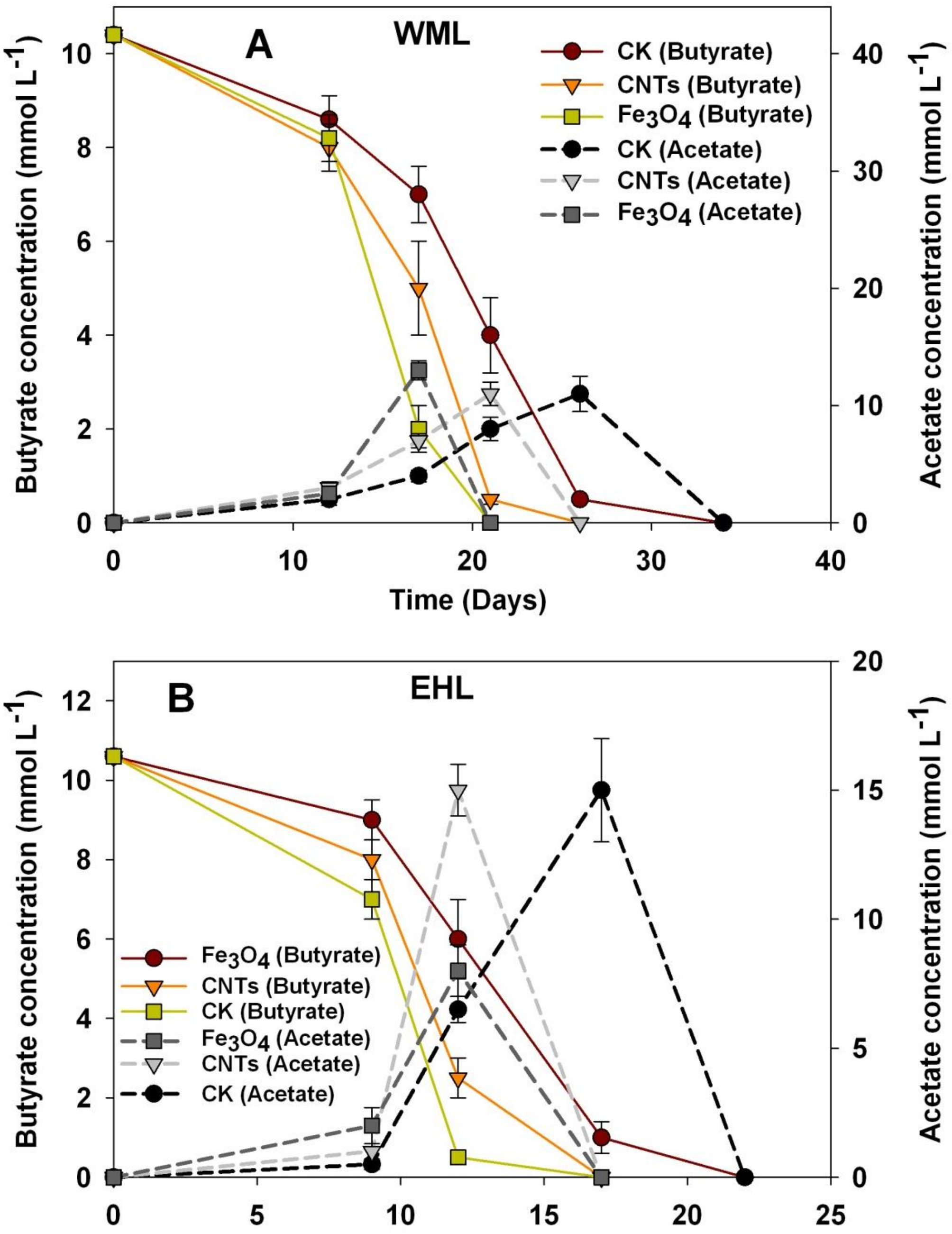

Supplementary figure 2. Butyrate oxidation and acetate formation in the fourth enrichment cultures with or without nanomaterial supplementation from WML (A) and EHL (B)

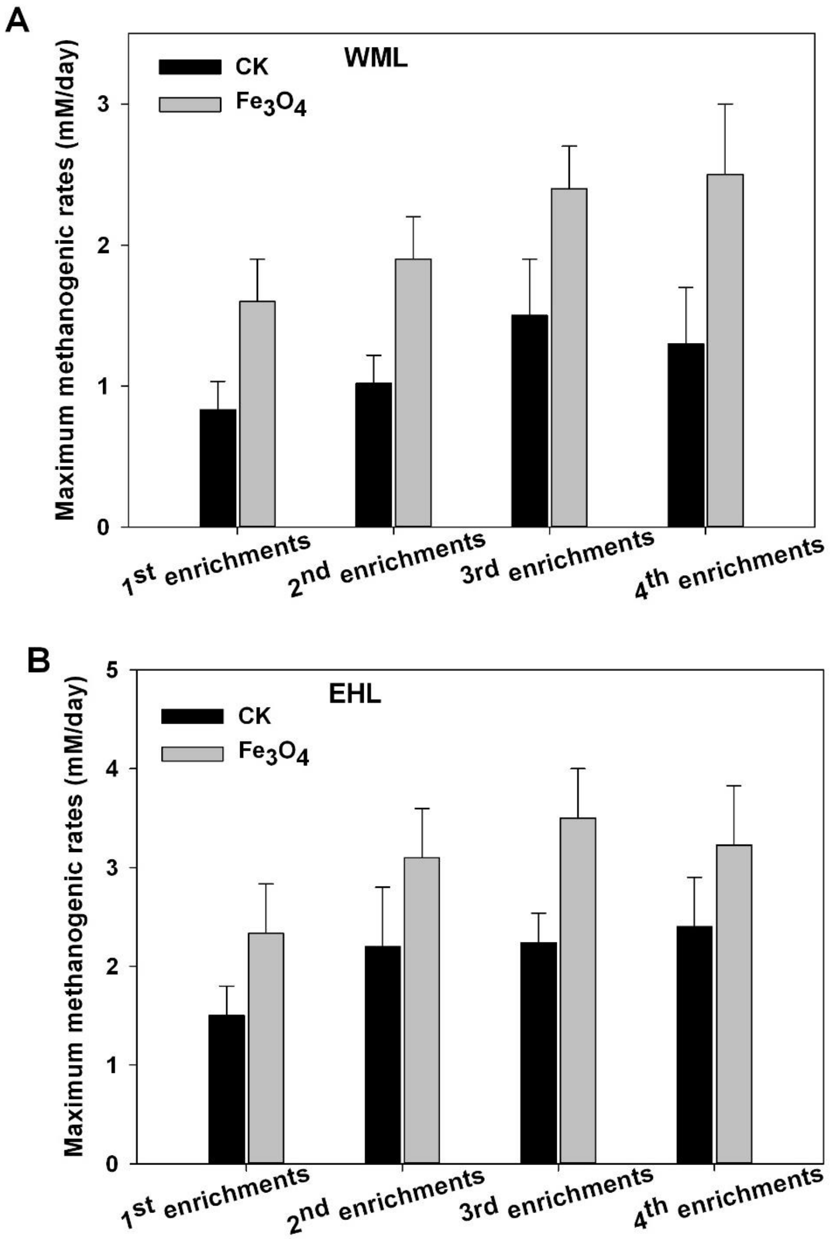

Supplementary figure 3. Effects of nanoFe_3O_4 on the maximum methanogenic rates of enrichment cultures in WML (A) and EHL (B)

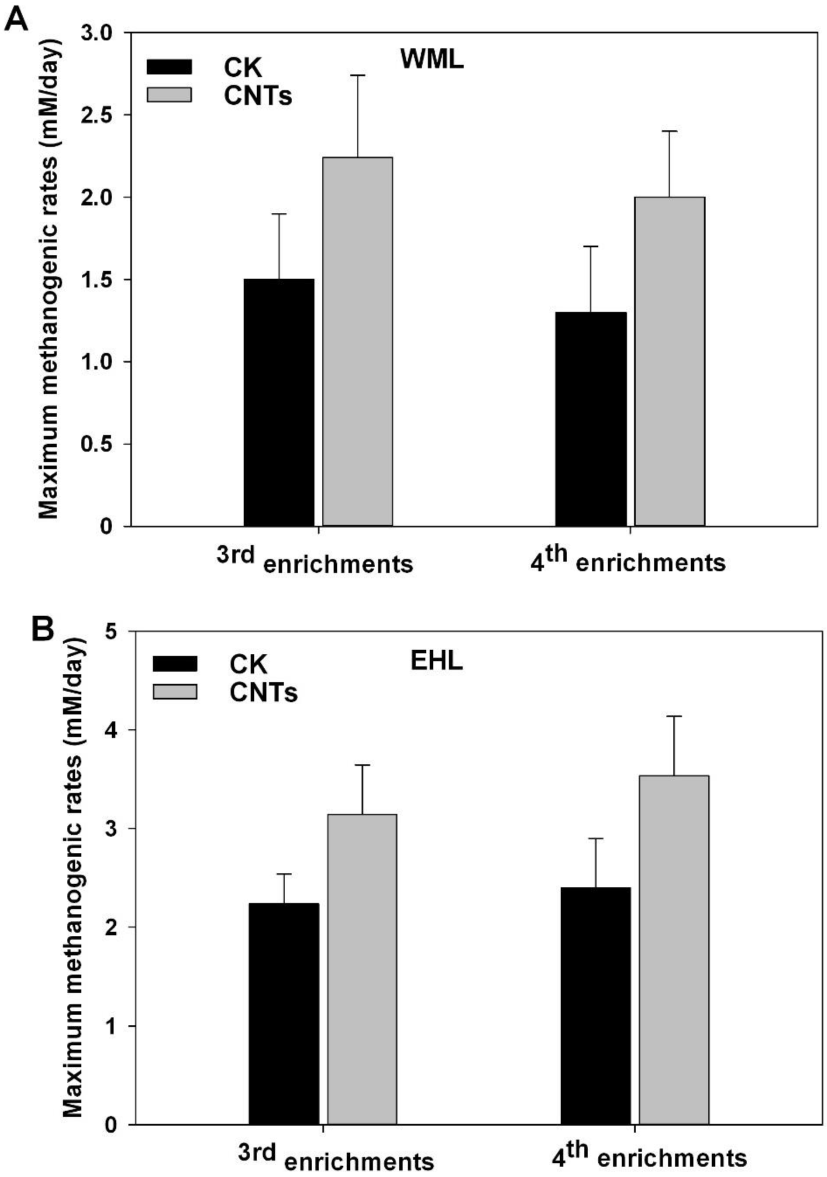

Supplementary figure 4. Effects of MWCNTs on the maximum methanogenic rates of enrichment cultures in WML (A) and EHL (B)
